# First genetic analysis of aneurysm genes in familial and sporadic abdominal aortic aneurysm

**DOI:** 10.1007/s00439-015-1567-0

**Published:** 2015-05-28

**Authors:** Koen M. van de Luijtgaarden, Daphne Heijsman, Alessandra Maugeri, Marjan M. Weiss, Hence J. M. Verhagen, Arne IJpma, Hennie T. Brüggenwirth, Danielle Majoor-Krakauer

**Affiliations:** Department of Vascular Surgery, Erasmus University Medical Center, Rotterdam, The Netherlands; Department of Anesthesiology, Erasmus University Medical Center, Rotterdam, The Netherlands; Department of Clinical Genetics, Suite-EE 2036, Erasmus University Medical Center, PO Box 2040, 3000 CA Rotterdam, The Netherlands; Department of Clinical Genetics, VU University Medical Center, Amsterdam, The Netherlands; Department of Bioinformatics, Erasmus University Medical Center, Rotterdam, The Netherlands

## Abstract

Genetic causes for abdominal aortic aneurysm (AAA) have not been identified and the role of genes associated with familial thoracic aneurysms in AAA has not been explored. We analyzed nine genes associated with familial thoracic aortic aneurysms, the vascular Ehlers–Danlos gene *COL3A1* and the *MTHFR* p.Ala222Val variant in 155 AAA patients. The thoracic aneurysm genes selected for this study were the transforming growth factor-beta pathway genes *EFEMP2, FBN1, SMAD3, TGBF2, TGFBR1, TGFBR2,* and the smooth muscle cells genes *ACTA2, MYH11* and *MYLK*. Sanger sequencing of all coding exons and exon–intron boundaries of these genes was performed. Patients with at least one first-degree relative with an aortic aneurysm were classified as familial AAA (*n* = 99), the others as sporadic AAA. We found 47 different rare heterozygous variants in eight genes: two pathogenic, one likely pathogenic, twenty-one variants of unknown significance (VUS) and twenty-three unlikely pathogenic variants. In familial AAA we found one pathogenic and segregating variant (*COL3A1* p.Arg491X), one likely pathogenic and segregating (*MYH11* p.Arg254Cys), and fifteen VUS. In sporadic patients we found one pathogenic (*TGFBR2* p.Ile525Phefs*18) and seven VUS. Thirteen patients had two or more variants. These results show a previously unknown association and overlapping genetic defects between AAA and familial thoracic aneurysms, indicating that genetic testing may help to identify the cause of familial and sporadic AAA. In this view, genetic testing of these genes specifically or in a genome-wide approach may help to identify the cause of familial and sporadic AAA.

**Electronic supplementary material:**

The online version of this article (doi:10.1007/s00439-015-1567-0) contains supplementary material, which is available to authorized users.

## Introduction

Approximately 20 % of the patients with an abdominal aortic aneurysm (AAA) have a positive family history for aneurysms, suggesting a genetic predisposition for AAA in these families (Rossaak et al. 2001; Salo et al. 1999; van de Luijtgaarden et al. 2014). The genetic aortic aneurysm syndromes Marfan, Loeys–Dietz, and aneurysms-osteoarthritis (AOS), involving the *FBN1*, *TGFBR1*, *TGFBR2*, *TGFB2*, and *SMAD3* genes were first identified in patients with pathologic dilatation or aneurysm of the thoracic aorta with multisystem overlapping cardiovascular, skeletal and ocular manifestations (Boileau et al. 2012; Cook et al. 2014; Dietz et al. 1991; Loeys et al. 2006; van de Laar et al. 2011). The genetic defects in these syndromes affect the integrity of the elastic medial layer by inference with the transforming growth factor-beta (TGF-β) pathway (Boileau et al. 2012; Judge and Dietz 2005; Lindsay et al. 2012; Loeys et al. 2005; ten Dijke and Arthur 2007). The wide range of variably expressed features in these rare autosomal dominantly inherited syndromic forms of familial thoracic aneurysm includes pectus- and/or spinal deformities, joint laxity, and skin translucency and specifically for AOS, osteoarthritis and for the Loeys–Dietz syndrome, hypertelorism, bifid uvula or cleft palate and arterial tortuosity. Vascular tortuosity, ascending aortic aneurysm, joint laxity and pectus excavatum are also main features of the *EFEMP2*-related autosomal recessive juvenile cutis laxa syndrome (Hucthagowder et al. 2006; Kappanayil et al. 2012).

In another group of families with thoracic aneurysm without distinct clinical features, genetic defects were identified in the so-called non-syndromic familial thoracic aneurysm genes including the *MYH11*, *MYLK* and *ACTA2* that affect smooth muscle cell (SMC) functioning (Kuang et al. 2012; Pannu et al. 2007; Renard et al. 2013; Wang et al. 2010). These may also affect TGF-β signaling, like *ACTA2* mutations, occurring in 16 % of patients with familial thoracic aortic aneurysm and in sporadic thoracic aortic aneurysms and dissections associated with medial degeneration, focal medial smooth muscle cell hyperplasia and proliferation, and stenotic arteries in the vaso-vasorum (Guo et al. 2007; Morisaki et al. 2009; Renard et al. 2013). A recent review estimates that approximately 20 % of familial thoracic aneurysm cases could be explained by a mutation in one of the thoracic aneurysm genes (Pomianowski and Elefteriades 2013). Establishing the exact contribution of each of these genes in (familial) thoracic aneurysms has been hampered by the overlap in clinical features. Occasionally isolated abdominal aortic aneurysms have been observed in families with familial syndromic and non-syndromic thoracic aneurysm. Therefore, genes associated with the familial thoracic aortic aneurysm may play a role in the degenerative changes of the extracellular matrix of the abdominal aortic wall underlying the formation of AAA. For this reason, we decided to screen AAA patients for variants in the transforming growth factor-beta pathway genes *EFEMP2, FBN1, SMAD3, TGBF2, TGFBR1*, *TGFBR2,* smooth muscle cells genes *ACTA2, MYH11* and *MYLK,* as well as the vascular Ehlers–Danlos gene *COL3A1*, which is associated with vascular fragility (Pepin et al. 2000). In addition, we investigated the previously reported association between abdominal aneurysm and the c.665C>T variant in *MTHFR* (Thompson et al. 2008). We report all the variants found in these analyses, except those classified as clearly not pathogenic (benign). The presented description of variants will convey relevance for classification of variants in future diagnostic setting.

## Materials and methods

The study complied with the declaration of Helsinki and was approved by the Institutional Review Board (MEC-2013-265).

### Study population

The study population consisted of 155 AAA patients referred for genetic counseling between January 2009 and December 2013 to the Department of Clinical Genetics at the Erasmus University Medical Center in Rotterdam, the Netherlands. Abdominal aortic aneurysm was defined as an external infrarenal abdominal aortic diameter ≥30 mm (Moll et al. 2011). Patients were classified as familial AAA when at least one first-degree relative (i.e., parent, sibling or child) was confirmed by medical records to be diagnosed with an aortic aneurysm (*n* = 99, 81 male). Patients reporting only affected second- or third-degree relatives were also classified as sporadic AAA, because the reporting of medical information of second- or third-degree relatives was considered less reliable (Andreasen et al. 1977). Patients without an affected first-degree relative were classified as sporadic AAA (*n* = 56, 46 male). In case of familial AAA, the first family member diagnosed with AAA was included as index in the study. Cases of concordant twins were considered as familial AAA. Genetic evaluation of the AAA patients was performed by a clinical geneticist and included ascertainment of a detailed family history and physical examination. All patients consented to DNA testing.

### DNA analysis and classification of variants

Sanger sequencing of all coding exons and exon–intron boundaries in *ACTA2* (NM_001613.1)*, COL3A1* (NM_000090.3), *EFEMP2* (NM_016938.3), *FBN1* (NM_000138.3)*, MYH11* (NM_001040113.1)*, MYLK* (NM_053025.3)*, SMAD3* (NM_005902.3), *TGFB2* (NM_001135599.2), *TGFBR1* (NM_004612.2) and *TGFBR2* (NM_001024847.2) was performed at the certified laboratories of the Departments of Clinical Genetics of the Erasmus University Medical Center in Rotterdam and the VU Medical Center in Amsterdam. Patients were tested for the p.Ala222Val variant in *MTHFR* (NM_005957.4) at the Department of Clinical Genetics at the University Hospital in Nijmegen, The Netherlands.

Assessment of the pathogenic effect of genetic variants was performed according to the guidelines currently used in the Rotterdam laboratory for DNA diagnostics with the use of Alamut Interactive Biosoftware (Rouen, France). This software incorporates SpliceSiteFinder-like, MaxEntScan, NNSPLICE, GeneSplicer, and Human Splicing Finder for the prediction of splicing variants and the programs Align GVGD, SIFT, Mutation Taster, PolyPhen-2 and KDv4 for in silico prediction of the effect of amino acid changes. Additionally, it gives population frequencies for dbSNP and ESP, and shows whether or not a variant has been reported before in Human Gene Mutation Database (HGMD). The Rotterdam classification system of variants was adapted from the sequence variation classification proposed by Plon et al. (2008). The criteria for classification of variants included the allele frequency in the dbSNP/ESP (cutoff 0.01), predicted effects on splicing, the in silico prediction of effect on the protein and previously described links to disease. Exonic variants remote from wild-type splice donor and acceptor sites were assessed to have no effect on splicing. For each variant present in HGMD the supporting evidence was reviewed and we evaluated whether previous reports linking specific variants to aneurysm were supported by functional studies or expression assays. Additionally, a variant only predicted by in silico prediction to be pathogenic would not automatically be classified as such because of lack of functional evidence. This resulted in categorizing variants into five classes: pathogenic, likely pathogenic, unknown significance (VUS), likely benign, and benign (Table 1; Richards et al. 2015). All variants expect those classified as benign were reported in this paper. A single previous description of a variant in a patient was not considered as sufficient evidence for causation and these variants were classified as variants of unknown significance instead of likely pathogenic. In addition, Table 2 presents the allele frequencies of the variants in the Dutch population derived from the GoNL cohort which contains data from parent–child combinations (Genome of the Netherlands 2014). From this source, only the information from the parents (*n* = 499) was used to compare the minor allele frequency (MAF) in the Dutch population to the allele frequencies derived from Alamut. We choose to add this information because the study population was predominantly (≥95 %) of Dutch ancestry and population-specific allele frequencies may help categorization of variants. Familial segregation of the variants with aneurysms in families was examined when affected relatives were available and consented for DNA testing.

**Table 1:** Classification of variants

Class	Variant	
Benign	Frequency in population ≥0.01	
Likely benign	Intronic or silent variants with no effect on splicing, missense variants for which 4/5 or 3/4 in silico protein predictions are neutral	
Unknown significance	Intronic, silent or missense variants that affect splicing, in-frame deletions/insertions, missense variants for which more than 2 in silico protein predictions are damaging	
Likely pathogenic	Frameshift, nonsense or intronic variants that affect splicing in a way that a new in-frame protein is created, missense variants that have previously been linked to disease in HGMD	
Pathogenic	Frameshift, nonsense or intronic variants that affect splicing in a way that no in-frame protein can be created

**Table 2:** Variants in familial and sporadic abdominal aortic aneurysm

Gene/variant	Protein change	dbSNP ID	MAF/MAC	Splice site prediction	Predicted pathogenic effect on protein (in silico,* n* programs)	Amino acid conservation (*n* species)	Classification	Number of variants (familial/sporadic AAA) (99/56)	Segregation^a^	MAF GoNL^b^
***COL3A1***								***3 (3/0)***		
c.812G>A (Pickup and Pollanen 2011)^c^	p.Arg271Gln	rs112185887	0.001/1	No effect	2/5	Moderately conserved (9)	VUS	1 (1/0)	nd	0.004
c.898-14A>G	Intronic	–	–	Effect	–	–	VUS	1 (1/0)	nd	na
** c.1471C>T**	**p.Arg491X**	**–**	**–**	**No effect**	**–**	**–**	**P**	**1 (1/0)**	**+**	**na**
***EFEMP2***								***6 (6/0)***		
c.160+17G>T	Intronic	–	–	No effect	–	–	LB	1 (1/0)	nd	na
c.277G>A^c^	p.Gly93Ser	rs2234462	0.001/3	No effect	0/4	Moderately conserved (10)	LB	3 (3/0)	−/nd	0.005
c.368-4G>A	Intronic	rs111550973	0.002/4	No effect	–	–	LB	1 (1/0)	−	0.007
c.1047C>T^c^	no-change	–	–	No effect	–	–	LB	1 (1/0)	nd	na
***FBN1***								***8 (5/3)***		
c.59A>G (Arbustini et al. 2005)^c,d^	p.Tyr20Cys	rs201309310	–	No effect	0/4	Moderately conserved (11)	VUS	1 (0/1)		na
c.248-17C>G^c^	Intronic	–	–	No effect	–	–	LB	1 (1/0)	−	na
c.1108G>A^c^	p.Val370lle	–	–	No effect	0/5	moderately conserved (11)	LB	1 (1/0)	nd	na
c.2260T>C^c^	p.Tyr754His	–	–	No effect	4/5	highly conserved (11)	VUS	1 (1/0)	nd	na
c.2895G > A^#^	no-change	rs140591	–	No effect	–	–	LB	1 (1/0)	nd	na
c.3455C>T (Hung et al. 2009)^d^	p.Ala1152Val	–	–	Effect	1/5	Highly conserved (11)	VUS	1 (0/1)		na
c.6055G>A (Sheikhzadeh et al. 2012)^d^	p.Glu2019Lys	–	–	No effect	3/5	Highly conserved (11)	VUS	1 (1/0)	nd	0.001
c.7412C>G	p.Pro2471Arg	rs193922233	–	No effect	3/5	Highly conserved (11)	VUS	1 (0/1)		na
***MYH11***								***11 (9/2)***		
** c.760C>T** (Kuang et al. 2012)^a^	**p.Arg254Cys**	**rs150759461**	**0.001/2**	**No effect**	**3/4**	**Highly conserved (13)**	**LP**	**1 (1/0)**	**+**	**0.008**
c.956A>G	p.Asn319Ser	rs149964928	–	Effect	1/4	Highly conserved (13)	VUS	1 (1/0)	−	na
c.1523G>A	p.Arg508His	rs144244239	0.001/2	No effect	3/4	Highly conserved (13)	VUS	1 (1/0)	nd	na
c.1868C>G	p.Ala623Gly	rs140688587	–	No effect	1/4	highly conserved (13)	LB	1 (1/0)	−	na
c.2881-14C>G	Intronic	–	–	No effect	–	–	LB	1 (1/0)	+	na
c.4694C>T^c^	p.Thr1565Met	rs111854563	0.001/1	No effect	3/4	Moderately conserved (13)	VUS	1 (1/0)	gem	0.004
c.5587C>T^c^	no-change	rs142639688	–	No effect	–	–	LB	1 (1/0)	nd	0.004
c.5635-7G>A^c^	Intronic	rs202120792	0.001/1	No effect	–	–	LB	2 (1/1)	nd	na
c.5697G>C^c^	p.Glu1899Asp	rs113964173	0.005/10	No effect	4/4	Highly conserved (13)	VUS	1 (1/0)	−	0.008
c.5808-11_-8del	Intronic	–	–	Effect	–	–	VUS	1 (0/1)		na
***MYLK***								***19 (13/6)***		
c.312T>C	no-change	rs147597398	–	No effect	–	–	LB	1 (1/0)	gem	na
c.745T>G	p.Ser249Ala	–	–	No effect	2/5	Highly conserved (7)	VUS	1 (0/1)		na
c.1314C > T	no-change	rs200423954	0.001/2	No effect	–	–	LB	1 (0/1)		0.001
c.1327C>T^c^	p.Pro443Ser	rs35156360	0.006/13	No effect	4/5	Highly conserved (7)	VUS	4 (4/0)	-/nd	0.014
c.2101G > A	p.Ala701Thr	rs142835596	0.003/7	No effect	2/5	Highly conserved (7)	VUS	1 (0/1)		na
c.3184G>T^c^	p.Ala1062Ser	rs11558550	–	No effect	0/5	Highly conserved (7)	LB	1 (0/1)		na
c.3302A>G^#^	p.Lys1101Arg	–	–	No effect	0/5	Highly conserved (7)	LB	1 (1/0)	−	na
c.3403G>A	p.Gly1135Arg	–	–	No effect	4/5	Highly conserved (7)	VUS	1 (1/0)	+	na
c.3583A>G^c^	p.Asn1195Asp	–	–	No effect	3/5	Highly conserved (7)	VUS	1 (1/0)	nd	na
c.4179C>T	no-change	–	–	No effect	–	–	LB	2 (1/1)	−	na
c.4764G>A^c^	no-change	rs56056823	0.003/6	No effect	–	–	LB	3 (2/1)	nd	0.008
c.4785C>T	no-change	–	–	No effect	–	–	LB	1 (1/0)	nd	na
c.5079G>A^c^	no-change	rs141467675	0.002/5	No effect	–	–	LB	1 (1/0)	nd	na
***TGFB2***								***2 (2/0)***		
c.272G>A^c^	p.Arg91His	rs10482721	0.001/3	No effect	3/4	Highly conserved (12)	VUS	1 (1/0)	nd	0.005
c.703G>C	p.Val235Leu	rs10482810	0.001/2	No effect	2/4	highly conserved (12)	VUS	1 (1/0)	nd	0.006
***TGFBR1***								***5 (4/1)***		
c.15C>T^c^	no-change	–	–	No effect	–	–	LB	1 (1/0)	nd	na
c.214A>T	p.Ile72Leu	rs111513627	–	No effect	2/5	Highly conserved (12)	VUS	2 (1/1)	−	na
c.927G>C^c^	no-change	–	–	No effect	–	–	LB	1 (1/0)	nd	na
c.1125A>C^c^	no-change	rs7861780	0.003/6	No effect	–	–	LB	1 (1/0)	nd	0.004
***TGFBR2***								***3 (2/1)***		
c.1137C>T	no-change	rs113194608	–	No effect	–	–	LB	1 (1/0)	+	0.004
c.1234G>A (Matyas et al. 2006)^c,d^	p.Val412Met	rs35766612	0.001/1	No effect	3/4	Highly conserved (14)	VUS	1 (1/0)	gem	0.002
** c.1573delA** ^**c**^	p.Ile525Phefs*18	**–**	**–**	**No effect**	**–**	**–**	**P**	**1 (0/1)**		**na**

In bold the variants classified pathogenic or likely pathogenic

*P* pathogenic, *LP* likely pathogenic, *VUS* variant of unknown clinical significance, *LB* likely benign, *MAF* minor allele frequency, *MAC* minor allele count, *na* not applicable

^a^ For familial AAA, segregation of the variant in affected relatives was investigated, (+) variant present in all affected relatives in one family, (−) variant did not segregate in a family with AAA, (nd) segregation of the variant in familial AAA was not determined, (gem) affected gemelli with variant

^b^ Frequency in GoNL based on 499 studied individuals, 998 alleles, (na) variant was not reported in GoNL data

^c^ Variant is involved in complex genotypes

^d^ Variant reported in aneurysm patient in HGMD

## Results

Forty-seven variants were detected in 31 familial AAA (31 %) patients and 12 sporadic AAA (21 %) patients in *COL3A1, EFEMP2, FBN1, MYH11, MYLK*, *TGBF2, TGFBR1*, and *TGFBR2*, no variants were found in *ACTA2* and *SMAD3* (Table 2).

### Pathogenic variants

Two variants were classified as pathogenic. A *COL3A1* null mutation p.Arg491X was observed, segregating in patients with aneurysms in one family. This null mutation was observed in a 49-year-old man diagnosed with a small dissection of the arteria lienalis at screening for familial abdominal aneurysms (Supplementary online table). His paternal aunt who had a successful repair of an infrarenal aneurysm at age 69 also had the mutation. Her brother was reported with a sudden death at age 32 years. No autopsy was performed. Screening for the *COL3A1* mutation detected one asymptomatic 50-year-old female carrier without signs of vascular pathology on computed tomography angiography. None of the carriers of the null mutations showed distinct loss of subcutaneous fat, skin fragility, abnormal scarring or suffered from complications or bleeding after surgery or childbirth.

A novel heterozygous single base pair deletion in *TGFBR2*, p.Ile525Phefs*18 was found de novo in a 47-year-old male presenting with complex vascular pathology. This patient presented at the emergency room with severe acute abdominal pain and was diagnosed with a Stanford type-B aortic dissection associated with a pre-existing large aorto-iliac aneurysm and marked aorta-iliacal aneurysm with tortuosity with a diameter of 98.5 mm. The ascending aortic diameter was normal measuring 32.0 mm. The patient was treated with β-blockade and blood pressure control, resulting in complete remission of symptoms. Two weeks after onset of the symptoms, the aorto-iliac aneurysm was successfully repaired with an aorto-bifemoral Dacron bypass (van de Luijtgaarden et al. 2013). Marked arterial elongation and tortuosity of the abdominal aorta and iliac arteries was present without characteristic facial or musculo-skeletal signs of the Loeys–Dietz or Marfan syndrome. DNA analysis of his parents showed that the mutation occurred de novo and no other relatives were affected. This patient also had a likely benign *MYH11* intronic variant c.5635-7G > A just before exon 40. The likely benign *MYH11* variant occurred also in his two unaffected sisters and their 74-year-old unaffected father.

### Likely pathogenic variants

The missense variant in *MYH11* (p.Arg254Cys) was classified as likely pathogenic because a report showing pathogenic effects was available (Kuang et al. 2012). This variant was initially detected in a 73-year-old woman with a symptomatic abdominal aneurysm. The mutation was also present in her 44-year-old son diagnosed by family screening with bilateral aneurysm of the iliac arteries and a small abdominal aneurysm.

### Variants of unknown significance (VUS)

Twenty-one VUS included 19 missense and 2 intronic variants. In familial cases 15 VUS were observed; 14 missense and one intronic. In sporadic cases, 7 VUS were observed; 6 missense and one intronic. Two VUS were present in multiple patients. The *MYLK* (p.Pro443Ser) variant was found in four patients with familial AAA, but this variant did not segregate in one family and segregation could not be tested in the other families. The *TGFBR1* (p.Ile72Leu) variant was present in one sporadic and one familial case, and did not segregate. Three VUS variants in *FBN1* (p.Tyr20Cys, p.Ala1152Val, and p.Glu2019Lys) were previously reported in patients with Marfan syndrome, without sufficient evidence to be classified as likely pathogenic (Arbustini et al. 2005; Hung et al. 2009; Sheikhzadeh et al. 2012). The *TGFBR2* (p.Val412Met) variant in two affected twin brothers was previously reported in thoracic aneurysm (Matyas et al. 2006). The VUS missense variant in *COL3A1* (p.Arg271Gln) was previously linked to Ehlers–Danlos syndrome in the literature, however, after critical evaluation of the report, the evidence for this link was considered not sufficient to classify the variant as pathogenic (Pickup and Pollanen 2011).

### Likely benign variants (LB)

Of the twenty-three likely benign variants five were intronic, 13 were synonymous and 5 were missense variants. Four LB variants were observed in more than one patient: the synonymous *MYLK* c.4764G>A was present in three patients (two familial, one sporadic), the synonymous *MYLK* c.4179C>T and the intronic variant *MYH11* c.5635-7G>A occurred in one familial and one sporadic patient, and the missense *EFEMP2* p.Gly93ser was present in three patients with familial AAA without evidence of segregation.

### MTHFR c.665C>T

The *MTHFR* c.665C>T (p.Ala222Val) variant, previously reported as C677T, was tested in 130 patients (89 familial and 41 sporadic AAA patients). Twelve patients (9 %) were homozygous for the variant allele: ten (11 %) familial and two (5 %) sporadic. Forty-five (38 %) patients were heterozygous for the variant allele: thirty one (35 %) familial and fourteen (34 %) sporadic patients. The MAF in our study population was 0.265 compared to 0.320 in the Dutch GoNL cohort (Table 3).

**Table 3:** Frequencies of the *MTHFR* c.665C>T variant in AAA and control populations

References	Patients (*N*)	Diagnosis	MAF	Normal (CC)	Heterozygote (CT)	Homozygote (TT)	CT and TT
Brunelli et al. (2000)	58	AAA	0.483	14 (24 %)	32 (55 %)	12 (21 %)	44 (76 %)
	60	Control	0.392	19 (32 %)	35 (58 %)	6 (10 %)	41 (68 %)
Strauss et al. (2003)	63	AAA	0.365	21 (33 %)	38 (60 %)	4 (6 %)	42 (67 %)
	75	Control	0.231	49 (65 %)	20 (27 %)	6 (8 %)	26 (35 %)
Jones et al. (2005, 2013)	428	AAA^a^	0.310	211 (49 %)	169 (40 %)	48 (11 %)	217 (51 %)
	282	Control (healthy)	0.309	134 (48 %)	122 (43 %)	26 (9 %)	148 (52 %)
	271	Control (CVD)	0.303	137 (51 %)	104 (38 %)	30 (11 %)	134 (49 %)
	226	Control (PAD)	0.332	106 (47 %)	90 (40 %)	30 (13 %)	120 (53 %)
Sofi et al. (2005)	438	AAA	0.430	141 (32 %)	217 (50 %)	80 (18 %)	297 (68 %)
	438	Controls	0.380	166 (38 %)	211 (48 %)	61 (14 %)	272 (62 %)
Ferrara et al. (2006)	42	AAA > 60 years	0.298	18 (43 %)	23 (55 %)	1 (2 %)	24 (57 %)
	46	AAA < 60 years	0.424	10 (22 %)	33 (72 %)	3 (6 %)	36 (78 %)
	45	Control	0.133	34 (75 %)	10 (23 %)	1 (2 %)	11(24 %)
Current study	130	AAA total	0.265^a^	73 (56 %)	45 (35 %)	12 (9 %)	57 (44 %)
	89	AAA familial	0.287	48 (54 %)	31 (35 %)	10 (11 %)	41 (46 %)
	41	AAA sporadic	0.220	25 (61 %)	14 (34 %)	2 (5 %)	16 (39 %)
Overall	1205	AAA	0.364	488 (40 %)	557 (46 %)	160 (13 %)	717 (60 %)
	1397	Control	0.326	645 (46 %)	592 (42 %)	160 (11 %)	752 (54 %)

^a^MAF in Dutch population: 0.320

### Complex genotypes

Thirteen AAA patients (11 familial, including one pair of concordant monozygotic twins, and 2 sporadic) had two or more variants (Table 4). One pathogenic *TGFBR2* was involved in a complex genotype with a likely benign variant in *MYH11.*

**Table 4:** AAA patients with multiple variants in aneurysm genes

Patient	Familial/sporadic AAA	COL3A1	EFEMP2	FBN1	MTHFR	MYH11	MYLK	TGFB2	TGFBR1	TGFBR2
1.	Familial	–	–	LB c.1108G>A	–	–	–	–	LB c.15C > T	–
2.	Sporadic	–	–	VUS c.59A>G	–	–	LB c.3184G>T	–	–	–
3.	Familial	–	–	LB _c.2895G>A	–	–	VUS c.4764G>A	–	–	–
4.^b^	Familial	–	–	–	–	VUS c.4694C>T		–	–	VUS c.1234G>A
5.	Sporadic	–	–	–	–	LB c.5635-7G>A	–	–	–	**P c.1573delA** ^a^
6.	Familial	–	–	–	–	VUS c.5697G>C		–	LB c.1125A>C	–
7.	Familial	–	LB c.277G>A	–	–	–	LB c.3302A>G	–	–	–
8.^b^	Familial	VUS c.812G > A	–	–	–	–	VUS c.1327C>T	–	–	–
9.	Familial	–	LB c.277G>A	–	–	LB c.5587C>T	VUS c.1327C>T	–	–	–
10.	Familial	–	–	LB c.248-17C>G	–	VUS c.956A>G	–	–	–	–
11.	Familial	–	LB c.1047C>T and (3)c.277G>A	–	–	–	–	–	LB c.927G>C	–
12.^b^	Familial	–	–	VUS c.2260T>C	–	–	–	VUS c.272G>A	–	–
13.	Familial	–	–	–	–	–	LB c.3583A>G and VUS c.5079G>A	–	–	–

Variants of class 4 or higher are shown in bold

*P* pathogenic, *LP* likely pathogenic, *VUS* variant of unknown clinical significance, *LB* likely benign

^a^ de novo mutation

^b^ Patient with multiple variants of VUS or higher

### Frequency of variants in the Dutch population

For 16 of the variants found in this study, the allele frequency in the Dutch population was available from GoNL. The MAF of the VUS variant in *MYLK* (p.Pro443Ser) was 0.014 in the Dutch population, and was reported in dbSNP with an MAF of 0.006. This indicates that this variant would be reclassified as a benign variant, using the GoNL frequency information instead of the frequency reported by dbSNP/ESP used in Alamut.

## Discussion

The genetic defects causing familial abdominal aortic aneurysm are poorly understood. This study showed that genes known to be associated with inherited thoracic aortic aneurysm also have a role in abdominal aortic aneurysm. Our study is based on a group of AAA patients referred for counseling. Therefore, the observed results do not represent prevalence of variants in the Dutch AAA population. Although familial cases were overrepresented in the current study, a referral bias for genotype can be excluded, since there was no prior information on genetic defects in familial or sporadic AAA. The validation of our family history data showed that no relatives were reported incorrectly as affected, indicating that risk in relatives was not overestimated. On the other hand, underreporting of familial disease may have happened, in particular for a disease like AAA, where aneurysms in relatives may go unnoticed and relatives could have undiagnosed aneurysms or may have died before age of onset. It is, therefore, important to bear in mind that familial AAA cannot be excluded when family history of aneurysm is uninformative or missing.

This study investigated the association between AAA and the thoracic aneurysm genes *ACTA2*, *COL3A1, EFEMP2, FBN1, MYH11, MYLK*, *SMAD3*, *TGBF2, TGFBR1*, *TGFBR2,* and *MTHFR* (p.Ala222Val). There have been several large GWAS studies that found AAA risk alleles in *LRP1*, (Bown et al. 2011) *DAP21P*, (Gretarsdottir et al. 2010) *ANRIL*, (Helgadottir et al. 2008) and *SORT1* (Jones et al. 2013). These genes have not been tested in this study, but it would be useful to do so in future studies.

In this study, three variants were observed classified as pathogenic or likely pathogenic amongst a total of 47 unique rare variants in our AAA study population of 155 patients. Lack of a comprehensive overview of genetic variants in thoracic aneurysms precluded comparison of our findings in the abdominal aneurysm population to thoracic aneurysms population.

Assessment of pathogenicity of genetic variants remains a major challenge (MacArthur et al. 2014). Comprehensive guidelines are needed to distinguish true pathogenic from ambiguous variants with unknown clinical significance which constitutes a large part of the results of molecular analyses (Richards et al. 2015). Variants are listed in HGMD which reports whether variants and/or genes have been described in the literature as associated with disease requiring critical review of evidence presented to justify classification as likely pathogenic. Additional searches may be needed because not all known variants are listed in HGMD. Establishing a causal effect of variants involves finding a method of choice for functional testing of variants in aneurysm genes, which is complicated giving the likelihood of tissue-specific gene expression. Especially since nowadays abdominal aortic aneurysms are mostly restored by an endovascular procedure, no aortic aneurysm tissue from patients can be collected for functional testing.

Alamut incorporates allele frequency reported by dbSNP/ESP. The use of population-specific control cohorts, as GoNL in the current study, may improve correct classification of variants and prevent associating population-specific polymorphisms with disease.

Significant co-segregation of a variant with disease provides evidence to support pathogenicity. In our study population it was often not possible to detect co-segregation because AAA is a late onset disorder, where the majority of patients, familial AAA and sporadic AAA alike, are older than 65 years and most affected relatives are no longer alive. Although our results suggest that more variants occur in familial cases (31 %) than in sporadic cases (21 %), the available sample size of the study population did not provide sufficient statistical power to test the difference between familial and sporadic AAA (Table 5).

**Table 5:** Genetic variants in 99 familial and 56 sporadic abdominal aortic aneurysm patients

Gene/variants	Total AAA patients			Familial AAA patients			Sporadic AAA patients			*p* value	
	*n*	ALL	LP/P	*n*	ALL	LP/P	*n*	All	LP/P	ALL	LP/P
ACTA2	139	0	0	92	0	0	47	0	0	1	1
COL3A1	122	3 (2 %)	1 (1 %)	82	3 (2 %)	1 (1 %)	40	0	0	1	1
EFEMP2	89	5 (7 %)	0	65	5 (8 %)	0	24	0	0	0.19	1
FBN1	127	8 (6 %)	0	85	5 (6 %)	0	42	3 (7 %)	0	1	1
MYH11	133	11 (8 %)	1 (1 %)	90	9 (10 %)	1 (1 %)	43	2 (5 %)	0	0.50	1
MYLK	136	18 (13 %)	0	90	12 (13 %)	0	46	6 (13 %)	0	1	1
SMAD3	142	0	0	94	0	0	48	0	0	1	1
TGFB2	65	2 (3 %)	0	40	2 (5 %)	0	25	0	0	0.52	1
TGFBR1	141	5 (4 %)	0	93	4 (4 %)	0	48	1 (2 %)	0	0.66	1
TGFBR2	140	3 (2 %)	1 (1 %)	94	2 (2 %)	0	46	1 (2 %)	1 (2 %)	1	0.33

*LP* likely pathogenic, *P* pathogenic, All variants: pathogenic, likely pathogenic, unknown clinical significance and likely benign

* P value was calculated using the two-tailed Fisher’s exact test

### *COL3A1*

The vascular type of the Ehlers–Danlos syndrome has caused mutations in type III procollagen encoded by the *COL3A1* gene (Pepin et al. 2000). Abnormal type III procollagen results in altered connective tissue in particular of the vascular wall, skin and inner organs. The Ehlers–Danlos type IV syndrome is associated with vascular fragility, thin and translucent skin, typical facial features, rupture uterus or intestines, and variably hypermobility or contractures. In the *COL3A1* gene we found one familial pathogenic null mutation and two VUS variants in familial AAA. Null mutations in *COL3A1* cause haploinsufficiency and were previously associated with attenuated clinical features of the vascular type of Ehlers–Danlos syndrome, like in the family described in the current study (Leistritz et al. 2011).

### *EFEMP2*

No pathogenic *EFEMP2* variants were detected in AAA patients. Four LB variants were observed in five familial AAA patients (8 %). One patient had two different variants in *EFEMP2*. One LB variant with an allele frequency of 0.005 in the Dutch population occurred in three patients.

### *FBN1*

The Marfan syndrome (MFS) was the first well-recognized genetic aortic aneurysm syndrome described in 1896, (Marfan 1896) and has an estimated prevalence of 2–3 per 10,000 individuals equally affecting men and women (Pyeritz and McKusick 1979). We found five *FBN1* VUS, two in familial and three in sporadic patients including three VUS previously reported in aneurysm patients in HGMD (Table 2).

### *MYH11*

One likely pathogenic segregating variant and five VUS were observed among the ten rare variants in this gene.

### *MYLK*

The *MYLK* gene harbored the most variants of all examined genes in this study in familial and sporadic patients. We found 13 unique variants in 18 patients, in 13 % of the familial and 13 % of the sporadic AAA patients. Of these variants, five were VUS and the rest was classified as likely benign.

### *TGFB2*

Boileau and Lindsay et al. described simultaneously a mutation in the *TGFB2* gene causing familial thoracic aortic aneurysm and dissections and overlapping clinical features with Loeys–Dietz syndrome (Boileau et al. 2012; Lindsay et al. 2012). We observed two VUS in the *TGFB2* in two patients with familial AAA (6 %).

### *TGFBR1* and *TGFBR2*

In *TGFBR1* we found variants in 4 % of familial and 2 % of sporadic patients, one VUS and three likely benign. To our knowledge no pathogenic variants in *TGFBR1* or *TGFBR2* have been linked to familial AAA. Specific alleles were previously associated with risk for AAA (Baas et al. 2010; Lucarini et al. 2009).

In *TGFBR2,* we found one de novo pathogenic novel single base pair deletion leading to a truncated protein. We also found one missense variant that was reported in HGMD and was classified as VUS, and one variant that we classified as likely benign.

### *MTHFR*

The role in the susceptibility for abdominal aortic aneurysms of the *MTHFR* c.665C>T (p.Ala222Val) variant, previously reported as C677T, was investigated by a number of case–control studies showing more robust associations in some than in others (Narayanan et al. 2013). More recently, genome-wide association studies endorsed that this variant was associated with an increased risk (Brunelli et al. 2000; Ferrara et al. 2006; Jones et al. 2005; Sofi et al. 2005; Strauss et al. 2003). In the current study, the MAF of the risk allele was lower (0.265) than in the Dutch control population (0.320), indicating that our data did not support a link with AAA.

## Concluding remarks

This study identified three causal variants in a set of genes previously associated with familial thoracic aortic aneurysms in 155 familial and sporadic AAA patients. Our results showed that diagnostic testing of these aneurysm genes might help find the cause for AAA and help to accurately identify relatives at risk. It is important to note the occurrence of de novo mutations, indicating that a negative family history should not preclude genetic testing. Pathogenic variants were found in two younger male patients with complex vascular features and an elderly female AAA patient. Although we cannot exclude an effect of referral bias, these observations merit further studies addressing the question whether age, gender and clinical features define a risk profile for molecular defects in AAA patients.

The identification of 44 other variants in genes associated with hereditary thoracic aneurysms suggests a more important contribution of these genes in AAA than known before. We expect that additional aneurysm-associated genes will be detected in the future because the majority of familial AAA patients had no variants in the examined genes.

## Electronic supplementary material

Supplementary material 1 (DOCX 52 kb)

## References

[CR1] Andreasen NC, Endicott J, Spitzer RL, Winokur G (1977). The family history method using diagnostic criteria. Reliability and validity. Arch Gen Psychiatry.

[CR2] Arbustini E, Grasso M, Ansaldi S, Malattia C, Pilotto A, Porcu E, Disabella E, Marziliano N, Pisani A, Lanzarini L, Mannarino S, Larizza D, Mosconi M, Antoniazzi E, Zoia MC, Meloni G, Magrassi L, Brega A, Bedeschi MF, Torrente I, Mari F, Tavazzi L (2005). Identification of sixty-two novel and twelve known FBN1 mutations in eighty-one unrelated probands with Marfan syndrome and other fibrillinopathies. Hum Mutat.

[CR3] Baas AF, Medic J, van’t Slot R, de Kovel CG, Zhernakova A, Geelkerken RH, Kranendonk SE, van Sterkenburg SM, Grobbee DE, Boll AP, Wijmenga C, Blankensteijn JD, Ruigrok YM (2010). Association of the TGF-beta receptor genes with abdominal aortic aneurysm. Eur J Hum Genet.

[CR4] Boileau C, Guo DC, Hanna N, Regalado ES, Detaint D, Gong L, Varret M, Prakash SK, Li AH, d’Indy H, Braverman AC, Grandchamp B, Kwartler CS, Gouya L, Santos-Cortez RL, Abifadel M, Leal SM, Muti C, Shendure J, Gross MS, Rieder MJ, Vahanian A, Nickerson DA, Michel JB, Jondeau G, Milewicz DM (2012). TGFB2 mutations cause familial thoracic aortic aneurysms and dissections associated with mild systemic features of Marfan syndrome. Nat Genet.

[CR5] Bown MJ, Jones GT, Harrison SC, Wright BJ, Bumpstead S, Baas AF, Gretarsdottir S, Badger SA, Bradley DT, Burnand K, Child AH, Clough RE, Cockerill G, Hafez H, Scott DJ, Futers S, Johnson A, Sohrabi S, Smith A, Thompson MM, van Bockxmeer FM, Waltham M, Matthiasson SE, Thorleifsson G, Thorsteinsdottir U, Blankensteijn JD, Teijink JA, Wijmenga C, de Graaf J, Kiemeney LA, Assimes TL, McPherson R, Consortium CA, Global BC, Consortium D, Consortium V, Folkersen L, Franco-Cereceda A, Palmen J, Smith AJ, Sylvius N, Wild JB, Refstrup M, Edkins S, Gwilliam R, Hunt SE, Potter S, Lindholt JS, Frikke-Schmidt R, Tybjaerg-Hansen A, Hughes AE, Golledge J, Norman PE, van Rij A, Powell JT, Eriksson P, Stefansson K, Thompson JR, Humphries SE, Sayers RD, Deloukas P, Samani NJ (2011) Abdominal aortic aneurysm is associated with a variant in low-density lipoprotein receptor-related protein 1. Am J Hum Genet 89:619–27 10.1016/j.ajhg.2011.10.002 PMC3213391 22055160

[CR6] Brunelli T, Prisco D, Fedi S, Rogolino A, Farsi A, Marcucci R, Giusti B, Pratesi C, Pulli R, Gensini GF, Abbate R, Pepe G (2000). High prevalence of mild hyperhomocysteinemia in patients with abdominal aortic aneurysm. J Vasc Surg.

[CR7] Cook JR, Carta L, Galatioto J, Ramirez F (2014) Cardiovascular manifestations in Marfan syndrome and related diseases; multiple genes causing similar phenotypes. Clin Genet 10.1111/cge.12436 24867163

[CR8] Dietz HC, Cutting GR, Pyeritz RE, Maslen CL, Sakai LY, Corson GM, Puffenberger EG, Hamosh A, Nanthakumar EJ, Curristin SM (1991). Marfan syndrome caused by a recurrent de novo missense mutation in the fibrillin gene. Nature.

[CR9] Ferrara F, Novo S, Grimaudo S, Raimondi F, Meli F, Amato C, Amodeo G, Lo Presti R, Caimi G (2006). Methylenetetrahydrofolate reductase mutation in subjects with abdominal aortic aneurysm subdivided for age. Clin Hemorheol Microcirc.

[CR10] Genome of the Netherlands C (2014). Whole-genome sequence variation, population structure and demographic history of the Dutch population. Nat Genet.

[CR11] Gretarsdottir S, Baas AF, Thorleifsson G, Holm H, den Heijer M, de Vries JP, Kranendonk SE, Zeebregts CJ, van Sterkenburg SM, Geelkerken RH, van Rij AM, Williams MJ, Boll AP, Kostic JP, Jonasdottir A, Walters GB, Masson G, Sulem P, Saemundsdottir J, Mouy M, Magnusson KP, Tromp G, Elmore JR, Sakalihasan N, Limet R, Defraigne JO, Ferrell RE, Ronkainen A, Ruigrok YM, Wijmenga C, Grobbee DE, Shah SH, Granger CB, Quyyumi AA, Vaccarino V, Patel RS, Zafari AM, Levey AI, Austin H, Girelli D, Pignatti PF, Olivieri O, Martinelli N, Malerba G, Trabetti E, Becker LC, Becker DM, Reilly MP, Rader DJ, Mueller T, Dieplinger B, Haltmayer M, Urbonavicius S, Lindblad B, Gottsater A, Gaetani E, Pola R, Wells P, Rodger M, Forgie M, Langlois N, Corral J, Vicente V, Fontcuberta J, Espana F, Grarup N, Jorgensen T, Witte DR, Hansen T, Pedersen O, Aben KK, de Graaf J, Holewijn S, Folkersen L, Franco-Cereceda A, Eriksson P, Collier DA, Stefansson H, Steinthorsdottir V, Rafnar T, Valdimarsson EM, Magnadottir HB, Sveinbjornsdottir S, Olafsson I, Magnusson MK, Palmason R, Haraldsdottir V, Andersen K, Onundarson PT, Thorgeirsson G, Kiemeney LA, Powell JT, Carey DJ, Kuivaniemi H, Lindholt JS, Jones GT, Kong A, Blankensteijn JD, Matthiasson SE, Thorsteinsdottir U (2010). Genome-wide association study identifies a sequence variant within the DAB2IP gene conferring susceptibility to abdominal aortic aneurysm. Nat Genet.

[CR12] Guo DC, Pannu H, Tran-Fadulu V, Papke CL, Yu RK, Avidan N, Bourgeois S, Estrera AL, Safi HJ, Sparks E, Amor D, Ades L, McConnell V, Willoughby CE, Abuelo D, Willing M, Lewis RA, Kim DH, Scherer S, Tung PP, Ahn C, Buja LM, Raman CS, Shete SS, Milewicz DM (2007). Mutations in smooth muscle alpha-actin (ACTA2) lead to thoracic aortic aneurysms and dissections. Nat Genet.

[CR13] Helgadottir A, Thorleifsson G, Magnusson KP, Gretarsdottir S, Steinthorsdottir V, Manolescu A, Jones GT, Rinkel GJ, Blankensteijn JD, Ronkainen A, Jaaskelainen JE, Kyo Y, Lenk GM, Sakalihasan N, Kostulas K, Gottsater A, Flex A, Stefansson H, Hansen T, Andersen G, Weinsheimer S, Borch-Johnsen K, Jorgensen T, Shah SH, Quyyumi AA, Granger CB, Reilly MP, Austin H, Levey AI, Vaccarino V, Palsdottir E, Walters GB, Jonsdottir T, Snorradottir S, Magnusdottir D, Gudmundsson G, Ferrell RE, Sveinbjornsdottir S, Hernesniemi J, Niemela M, Limet R, Andersen K, Sigurdsson G, Benediktsson R, Verhoeven EL, Teijink JA, Grobbee DE, Rader DJ, Collier DA, Pedersen O, Pola R, Hillert J, Lindblad B, Valdimarsson EM, Magnadottir HB, Wijmenga C, Tromp G, Baas AF, Ruigrok YM, van Rij AM, Kuivaniemi H, Powell JT, Matthiasson SE, Gulcher JR, Thorgeirsson G, Kong A, Thorsteinsdottir U, Stefansson K (2008). The same sequence variant on 9p21 associates with myocardial infarction, abdominal aortic aneurysm and intracranial aneurysm. Nat Genet.

[CR14] Hucthagowder V, Sausgruber N, Kim KH, Angle B, Marmorstein LY, Urban Z (2006). Fibulin-4: a novel gene for an autosomal recessive cutis laxa syndrome. Am J Hum Genet.

[CR15] Hung CC, Lin SY, Lee CN, Cheng HY, Lin SP, Chen MR, Chen CP, Chang CH, Lin CY, Yu CC, Chiu HH, Cheng WF, Ho HN, Niu DM, Su YN (2009). Mutation spectrum of the fibrillin-1 (FBN1) gene in Taiwanese patients with Marfan syndrome. Ann Hum Genet.

[CR16] Jones GT, Harris EL, Phillips LV, van Rij AM (2005). The methylenetetrahydrofolate reductase C677T polymorphism does not associate with susceptibility to abdominal aortic aneurysm. Eur J Vasc Endovasc Surg.

[CR17] Jones GT, Bown MJ, Gretarsdottir S, Romaine SP, Helgadottir A, Yu G, Tromp G, Norman PE, Jin C, Baas AF, Blankensteijn JD, Kullo IJ, Phillips LV, Williams MJ, Topless R, Merriman TR, Vasudevan TM, Lewis DR, Blair RD, Hill AA, Sayers RD, Powell JT, Deloukas P, Thorleifsson G, Matthiasson SE, Thorsteinsdottir U, Golledge J, Ariens RA, Johnson A, Sohrabi S, Scott DJ, Carey DJ, Erdman R, Elmore JR, Kuivaniemi H, Samani NJ, Stefansson K, van Rij AM (2013). A sequence variant associated with sortilin-1 (SORT1) on 1p13.3 is independently associated with abdominal aortic aneurysm. Hum Mol Genet.

[CR18] Judge DP, Dietz HC (2005). Marfan’s syndrome. Lancet.

[CR19] Kappanayil M, Nampoothiri S, Kannan R, Renard M, Coucke P, Malfait F, Menon S, Ravindran HK, Kurup R, Faiyaz-Ul-Haque M, Kumar K, De Paepe A (2012). Characterization of a distinct lethal arteriopathy syndrome in twenty-two infants associated with an identical, novel mutation in FBLN4 gene, confirms fibulin-4 as a critical determinant of human vascular elastogenesis. Orphanet J Rare Dis.

[CR20] Kuang SQ, Kwartler CS, Byanova KL, Pham J, Gong L, Prakash SK, Huang J, Kamm KE, Stull JT, Sweeney HL, Milewicz DM (2012). Rare, nonsynonymous variant in the smooth muscle-specific isoform of myosin heavy chain, MYH11, R247C, alters force generation in the aorta and phenotype of smooth muscle cells. Circ Res.

[CR21] Leistritz DF, Pepin MG, Schwarze U, Byers PH (2011). COL3A1 haploinsufficiency results in a variety of Ehlers-Danlos syndrome type IV with delayed onset of complications and longer life expectancy. Genet Med.

[CR22] Lindsay ME, Schepers D, Bolar NA, Doyle JJ, Gallo E, Fert-Bober J, Kempers MJ, Fishman EK, Chen Y, Myers L, Bjeda D, Oswald G, Elias AF, Levy HP, Anderlid BM, Yang MH, Bongers EM, Timmermans J, Braverman AC, Canham N, Mortier GR, Brunner HG, Byers PH, Van Eyk J, Van Laer L, Dietz HC, Loeys BL (2012). Loss-of-function mutations in TGFB2 cause a syndromic presentation of thoracic aortic aneurysm. Nat Genet.

[CR23] Loeys BL, Chen J, Neptune ER, Judge DP, Podowski M, Holm T, Meyers J, Leitch CC, Katsanis N, Sharifi N, Xu FL, Myers LA, Spevak PJ, Cameron DE, De Backer J, Hellemans J, Chen Y, Davis EC, Webb CL, Kress W, Coucke P, Rifkin DB, De Paepe AM, Dietz HC (2005). A syndrome of altered cardiovascular, craniofacial, neurocognitive and skeletal development caused by mutations in TGFBR1 or TGFBR2. Nat Genet.

[CR24] Loeys BL, Schwarze U, Holm T, Callewaert BL, Thomas GH, Pannu H, De Backer JF, Oswald GL, Symoens S, Manouvrier S, Roberts AE, Faravelli F, Greco MA, Pyeritz RE, Milewicz DM, Coucke PJ, Cameron DE, Braverman AC, Byers PH, De Paepe AM, Dietz HC (2006). Aneurysm syndromes caused by mutations in the TGF-beta receptor. N Engl J Med.

[CR25] Lucarini L, Sticchi E, Sofi F, Pratesi G, Pratesi C, Pulli R, Gensini GF, Abbate R, Pepe G, Fatini C (2009). ACE and TGFBR1 genes interact in influencing the susceptibility to abdominal aortic aneurysm. Atherosclerosis.

[CR26] MacArthur DG, Manolio TA, Dimmock DP, Rehm HL, Shendure J, Abecasis GR, Adams DR, Altman RB, Antonarakis SE, Ashley EA, Barrett JC, Biesecker LG, Conrad DF, Cooper GM, Cox NJ, Daly MJ, Gerstein MB, Goldstein DB, Hirschhorn JN, Leal SM, Pennacchio LA, Stamatoyannopoulos JA, Sunyaev SR, Valle D, Voight BF, Winckler W, Gunter C (2014). Guidelines for investigating causality of sequence variants in human disease. Nature.

[CR27] Marfan A (1896). Un cas de deformation congenitale des quatre membres plus prononcee aux extremites caracterisee par l’allongement des os avec un certain degre d’amincissement. Bull Mem Soc Med Hop Paris.

[CR28] Matyas G, Arnold E, Carrel T, Baumgartner D, Boileau C, Berger W, Steinmann B (2006). Identification and in silico analyses of novel TGFBR1 and TGFBR2 mutations in Marfan syndrome-related disorders. Hum Mutat.

[CR29] Moll FL, Powell JT, Fraedrich G, Verzini F, Haulon S, Waltham M, van Herwaarden JA, Holt PJ, van Keulen JW, Rantner B, Schlosser FJ, Setacci F, Ricco JB (2011). Management of abdominal aortic aneurysms clinical practice guidelines of the European society for vascular surgery. Eur J Vasc Endovasc Surg.

[CR30] Morisaki H, Akutsu K, Ogino H, Kondo N, Yamanaka I, Tsutsumi Y, Yoshimuta T, Okajima T, Matsuda H, Minatoya K, Sasaki H, Tanaka H, Ishibashi-Ueda H, Morisaki T (2009). Mutation of ACTA2 gene as an important cause of familial and nonfamilial nonsyndromatic thoracic aortic aneurysm and/or dissection (TAAD). Hum Mutat.

[CR31] Narayanan N, Tyagi N, Shah A, Pagni S, Tyagi SC (2013). Hyperhomocysteinemia during aortic aneurysm, a plausible role of epigenetics. Int J Physiol Pathophysiol Pharmacol.

[CR32] Pannu H, Tran-Fadulu V, Papke CL, Scherer S, Liu Y, Presley C, Guo D, Estrera AL, Safi HJ, Brasier AR, Vick GW, Marian AJ, Raman CS, Buja LM, Milewicz DM (2007). MYH11 mutations result in a distinct vascular pathology driven by insulin-like growth factor 1 and angiotensin II. Hum Mol Genet.

[CR33] Pepin M, Schwarze U, Superti-Furga A, Byers PH (2000). Clinical and genetic features of Ehlers-Danlos syndrome type IV, the vascular type. N Engl J Med.

[CR34] Pickup MJ, Pollanen MS (2011). Traumatic subarachnoid hemorrhage and the COL3A1 gene: emergence of a potential causal link. Forensic Sci Med Pathol.

[CR35] Plon SE, Eccles DM, Easton D, Foulkes WD, Genuardi M, Greenblatt MS, Hogervorst FB, Hoogerbrugge N, Spurdle AB, Tavtigian SV, Group IUGVW (2008). Sequence variant classification and reporting: recommendations for improving the interpretation of cancer susceptibility genetic test results. Hum Mutat.

[CR36] Pomianowski P, Elefteriades JA (2013). The genetics and genomics of thoracic aortic disease. Ann Cardiothorac Surg.

[CR37] Pyeritz RE, McKusick VA (1979). The Marfan syndrome: diagnosis and management. N Engl J Med.

[CR38] Renard M, Callewaert B, Baetens M, Campens L, MacDermot K, Fryns JP, Bonduelle M, Dietz HC, Gaspar IM, Cavaco D, Stattin EL, Schrander-Stumpel C, Coucke P, Loeys B, De Paepe A, De Backer J (2013). Novel MYH11 and ACTA2 mutations reveal a role for enhanced TGFbeta signaling in FTAAD. Int J Cardiol.

[CR39] Richards S, Aziz N, Bale S, Bick D, Das S, Gastier-Foster J, Grody WW, Hegde M, Lyon E, Spector E, Voelkerding K, Rehm HL (2015) Standards and guidelines for the interpretation of sequence variants: a joint consensus recommendation of the American College of Medical Genetics and Genomics and the Association for Molecular Pathology. Genet Med 10.1038/gim.2015.30 PMC4544753 25741868

[CR40] Rossaak JI, Hill TM, Jones GT, Phillips LV, Harris EL, van Rij AM (2001). Familial abdominal aortic aneurysms in the Otago region of New Zealand. Cardiovasc Surg.

[CR41] Salo JA, Soisalon-Soininen S, Bondestam S, Mattila PS (1999). Familial occurrence of abdominal aortic aneurysm. Ann Intern Med.

[CR42] Sheikhzadeh S, Kade C, Keyser B, Stuhrmann M, Arslan-Kirchner M, Rybczynski M, Bernhardt AM, Habermann CR, Hillebrand M, Mir T, Robinson PN, Berger J, Detter C, Blankenberg S, Schmidtke J, von Kodolitsch Y (2012). Analysis of phenotype and genotype information for the diagnosis of Marfan syndrome. Clin Genet.

[CR43] Sofi F, Marcucci R, Giusti B, Pratesi G, Lari B, Sestini I, Lo Sapio P, Pulli R, Pratesi C, Abbate R, Gensini GF (2005). High levels of homocysteine, lipoprotein (a) and plasminogen activator inhibitor-1 are present in patients with abdominal aortic aneurysm. Thromb Haemost.

[CR44] Strauss E, Waliszewski K, Gabriel M, Zapalski S, Pawlak AL (2003). Increased risk of the abdominal aortic aneurysm in carriers of the MTHFR 677T allele. J Appl Genet.

[CR45] ten Dijke P, Arthur HM (2007). Extracellular control of TGFbeta signalling in vascular development and disease. Nat Rev Mol Cell Biol.

[CR46] Thompson AR, Drenos F, Hafez H, Humphries SE (2008). Candidate gene association studies in abdominal aortic aneurysm disease: a review and meta-analysis. Eur J Vasc Endovasc Surg.

[CR47] van de Laar IM, Oldenburg RA, Pals G, Roos-Hesselink JW, de Graaf BM, Verhagen JM, Hoedemaekers YM, Willemsen R, Severijnen LA, Venselaar H, Vriend G, Pattynama PM, Collee M, Majoor-Krakauer D, Poldermans D, Frohn-Mulder IM, Micha D, Timmermans J, Hilhorst-Hofstee Y, Bierma-Zeinstra SM, Willems PJ, Kros JM, Oei EH, Oostra BA, Wessels MW, Bertoli-Avella AM (2011). Mutations in SMAD3 cause a syndromic form of aortic aneurysms and dissections with early-onset osteoarthritis. Nat Genet.

[CR48] van de Luijtgaarden KM, Bastos Goncalves F, Majoor-Krakauer D, Verhagen HJ (2013). Arterial elongation and tortuosity leads to detection of a de novo TGFBR2 mutation in a young patient with complex aortic pathology. Eur Heart J.

[CR49] van de Luijtgaarden KM, Bastos Goncalves F, Hoeks SE, Valentijn TM, Stolker RJ, Majoor-Krakauer D, Verhagen HJ, Rouwet EV (2014). Lower atherosclerotic burden in familial abdominal aortic aneurysm. J Vasc Surg.

[CR50] Wang L, Guo DC, Cao J, Gong L, Kamm KE, Regalado E, Li L, Shete S, He WQ, Zhu MS, Offermanns S, Gilchrist D, Elefteriades J, Stull JT, Milewicz DM (2010). Mutations in myosin light chain kinase cause familial aortic dissections. Am J Hum Genet.

