# Supplementary material for: First genetic analysis of aneurysm genes in familial and sporadic abdominal aortic aneurysm
Source: Hum Genet. 2015 May 28;134(8):881–93. doi: 10.1007/s00439-015-1567-0 (PMC4495250; doi:10.1007/s00439-015-1567-0)
Supplement: Supplementary file 1 — Supplementary material 1 (DOCX 52 kb) [file 439_2015_1567_MOESM1_ESM.docx]

|  | Supplementary Online Table  **Clinical features of abdominal aortic aneurysm patients with variants in aneurysm genes and the results of molecular an clinical family studies.** | | | | | | | | | | | |  |  | |
| --- | --- | --- | --- | --- | --- | --- | --- | --- | --- | --- | --- | --- | --- | --- | --- |
| **Gene/Variant** | | **Class*** | **Number of variants (familial/**  **sporadic AAA)**  **(99/56)** | **Segregation** | **Family #** | **Age (yrs)**  **index**  **(relatives)^r^** | **M/F** | **Aneurysm in**  **index and relatives** | **Body**  **length/**  **armspan ^+^**  **(ratio)^++^** | **Beighton**  **score** | | **Skin**  Extensible=1  Scarring=2**  Translucent=3  N=normal | **Skeletal**  Pectus excavatum=1  kyphoscoliosis=2  arthrosis=3  N= none | | **Other** |
| ***COL3A1*** | |  |  |  |  |  |  |  |  | |  |  |  | |  |
| c.812G>A^a^ | | VUS | 1 (1/0) | nd | 1/0 | 71  70^r^ | M  F | infrarenal ,dissection type B  infrarenal | -  - | | -  - | -  - | 3  - | |  |
| c.898-14A>G | | VUS | 1 (1/0) | nd | 5^b^/2^c^ | 67  60 ^r^  71 ^r^ | M  M  F | infrarenal,iliac  descendens, ascendens dilatation  descendens,iliac rupture | 178/188 ((1.05) | | 1  -  - | 3  1  - | 3  2  - | | retrognathia |
| **c.1471C>T** | | **P** | 1 (1/0) | **+** | 4/2 | 49  69 ^r^  59 ^r^ | M  F  F | art lienalis  infrarenal  infrarenal | 172/182 (1.05)  -  - | | 0  -  - | N  -  - | N  -  - | |  |
| ***EFEMP2*** | |  | ***6 (6/0)*** |  |  |  |  |  |  | |  |  |  | |  |
| c.160+17G>T | | LB | 1 (1/0) | nd | 5/2 | 68  72 ^r^  67 ^r^ | M  M  M | infrarenal  juxtarenal  infrarenal,iliac | 183/189 (1.03)  173/191 (1.10)  172/178 (1.03) | | 0  0  0 | 3  1  1,3 | 3  3  3 | | inguinal hernia |
| c.277G>A | | LB | 3 (3/0)^d^ | -  nd^f^  nd^e^ | 6/4  1/1  1/0 | 51  66 ^r^  48 ^r^  70 ^r^  55 ^r^  56  71 ^r^  72 | M  F  F  M  M  M  M  M | infrarenal,iliac  infrarenal  rupture infrarenal  rupture infrarenal  infrarenal  infrarenal  infrarenal  infrarenal | 177/182  -  -  -  -  176/185  -  - | | 6  -  -  -  -  5  -  3 | 2  -  -  -  -  1  -  N | N  -  -  -  -  N  -  N | |  |
| c.368-4G>A | | LB | 1 (1/0) | - | 6/5 | 69  69 ^r^  61 ^r^  43 ^r^  36 ^r^  71 ^r^ | M  F  M  F  F  F | infrarenal,dissection type B  ascendens  dissection type A  descendens dilatation  ascendens dilatation  descendens | 170/185 (1.08)  -  -  -  -  - | | 2  -  -  -  -  - | N  -  -  -  -  - | N  -  -  -  -  - | | wrist sign |
| c.1047C>T | | LB | 1 (1/0) | nd^f^ | 1/1 | 56  71 ^r^ | M  M | infrarenal  infrarenal | 176/185 (1.05)  - | | 5  - | 1  - | 3  - | | hypertelorism  inguinal hernia |
| ***FBN1*** | |  | ***8 (5/3)*** |  |  |  |  |  |  | |  |  |  | |  |
| c.59A>G^g^ | | VUS | 1 (0/1) |  |  | 72 | M | infrarenal | - | | 0 | N | N | | inguinal hernia |
| c.248-17C>G^h^ | | LB | 1 (1/0) | - | 1/1 | 65  69 ^r^ | M  F | infrarenal  infrarenal | 180/180  - | | 0  - | 2,3  - | 3  - | |  |
| c.1108G>A^i^ | | LB | 1 (1/0) | nd | 3/1 | 44  74 ^r^ | M  M | rupture infrarenal,iliac  infrarenal | 172/181 (1.05) | | 0  - | N  - | 3  - | |  |
| c.2260T>C ^j^ | | VUS | 1 (1/0) | nd | 1/0 | 58 | M | infrarenal,dilatation iliac | 171/175 | | 0 | N | N | |  |
| c.2895G>A^k^ | | LB | 1 (1/0) | nd | 3/0 | 50 | M | infrarenal | 173/173 | | 0 | 2,3 | N | |  |
| c.3455C>T | | VUS | 1 (0/1) |  |  | 61 | F | thoraco-abdominal | - | | - | - | - | |  |
| c.6055G>A | | VUS | 1 (1/0) | nd | 1/0 | 75 | F | infrarenal | 180/179 | | 6 | 1 | N | | wrist sign |
| c.7412C>G | | VUS | 1 (0/1) |  |  | 64 | M | infrarenal,iliac | 185/177 | | 7 | N | N | | inguinal hernia |
| ***MYH11*** | |  | ***11 (9/2)*** |  |  |  |  |  |  | |  |  |  | |  |
| **c.760C>T** | | LP | 1 (1/0) | **-** | 1/1 | 73  44 ^r^ | F  M | infrarenal  infrarenal,iliac | 173/179 (1.03)  197/199 | | 3  2 | N  2 | N  N | |  |
| c.956A>G^h^ | | VUS | 1 (1/0) | - | 1/1 | 65  69 ^r^ | M  F | infrarenal  infrarenal | 180/180  - | | 0  - | 2,3  - | 3  - | |  |
| c.1523G>A | | VUS | 1 (1/0) | nd | 1/0 | 77 | M | infrarenal | - | | - | - | - | |  |
| c.1868C>G | | LB | 1 (1/0) | - | 2/1 | 62  64 ^r^ | F  M | infrarenal  infrarenal | 171/169  - | | 9  - | N  - | N  - | | high palate,wrist sign |
| c.2881-14C>G | | LB | 1 (1/0) | - | 4^b^/1 | 63  62 | M  F | infrarenal  descendens | 185/186  - | | -  - | 2  - | -  - | |  |
| c.4694C>T ^l^ | | VUS | 1 (1/0) | gem | 1/1 | 60  61 | M  M | rupture infrarenal, iliac,popliteal  juxtarenal,,infrarenal,popliteal | 189/193  183/192 (1.04) | | 1  2 | 1,2,3  1,2 | 2 | | wrist sign |
| c.5587C>T^e^ | | LB | 1 (1/0) | nd | 1/0 | 72 | M | infrarenal | - | | 3 | N | N | |  |
| c.5635-7G>A | | LB | 2 (1/1) | nd | 1/1 | 48  54 ^r^  47 | F  M  M | rupture renal art  rupture renal art  infrarenal,iliac,dissection type B | 176/178  -  189/195 (1.03) | | 3  -  7 | 1  -  N | 2  -  3 | | high palatum  vascular tortuosity |
| c.5697G>C^m^ | | VUS | 1 (1/0) | - | 4/0 | 75 | F | infrarenal | 174/178 | | 1 | 2 | 3 | |  |
| c.5808-11-8del | | VUS | 1 (0/1) |  |  | 77 | M | rupture iliac,infrarenal | 175/178 | | 0 | N | N | | inguinal hernia |
| ***MYLK*** | |  | ***19 (13/6)*** |  |  |  |  |  |  | |  |  |  | |  |
| c.312T>C | | LB | 1 (1/0) | + | 1/1 | 49  50 | F  F | infrarenal, ascendens dilatation  infrarenal,iliac,popliteal | 175/188 (1,07)  178/191 (1,09) | | 4  9 | 2,3  2 | N  N | | wrist sign, thumb sign |
| c.745T>G | | VUS | 1 (0/1) |  |  | 92 | M | infrarenal | 192/195 | | 0 | 3 | 1 | | inguinal hernia |
| c.1314C>T | | LB | 1 (0/1) |  |  | 57 | M | rupture thoraco-abdominal,iliac | 176/177 | | 0 | 2,3 | 1 | |  |
| c.1327C>T | | VUS | 4 (4/0) | -  nd^e^  nd^a^  nd | 3/3  1/0  1/0  1/1 | 73  72 ^r^  77 ^r^  71 ^r^  72  71  70 ^r^  82  84 ^r^ | M  F  M  M  M  M  F  M  M | infrerenal  infrarenal,juxtarenal  infrarenal  infrarenal  infrarenal  infrarenal,dissection type B  infrarenal  infrarenal,iliac  infrarenal | 170/182 (1.07)  -  -  -  -  -  -  181/189 (1.04)  - | | 4  -  -  -  3  -  -  0  - | 2,3  -  -  2,3  N  -  -  2,3  - | N  N  N  3  -  3  - | | inguinal hernia  inguinal hernia |
| c.2101G>A | | VUS | 1 (0/1) |  |  | 74 | M | suprarenal, arteria renalis | 172/172 | | 8 | 1,2 | N | | gout |
| c.3184G>T^g^ | | LB | 1 (0/1) |  |  | 72 | M | infrarenal | - | | 0 | N | N | | inguinal hernia |
| c.3302A>G^d^ | | LB | 1 (1/0) | - | 6/4 | 51  66 ^r^  48 ^r^  70 ^r^  55 ^r^ | M  F  F  M  M | infrarenal,iliac  infrarenal  rupture infrarenal  rupture infrarenal  infrarenal | 177/182  -  -  -  - | | 6  -  -  -  - | 2  -  -  -  - | N  -  -  -  - | |  |
| c.3403G>A | | VUS | 1 (1/0) | + | 1/1 | 49  85 ^r^ | F  M | infrarenal  infrarenal,iliac | 185/181  - | | 9  - | N  - | 3  3 | | wrist sign |
| c.3583A>G^n^ | | VUS | 1 (1/0) | nd | 1/0 | 64 | F | infrarenal,iliac | 177/188 (1.06) | | 2 | N | 3 | |  |
| c.4179C>T | | LB | 2 (1/1) | nd | 1/0 | 74  67 | M  M | infrarenal  infrarenal | 181/189 (1.03)  - | | 9  - | 3  - | 2  - | |  |
| c.4764G>A^k^ | | LB | 3 (2/1) | nd  nd | 3/0  2/0 | 50  82  62 | M  M  M | infrarenal  infrarenal  thoraco- abdominal | 173/173  -  180/188 (1.04) | | 0  -  2 | 2,3  -  2 | N  -  1,2 | |  |
| c.4785C>T | | LB | 1 (1/0) | nd | 1/1 | 59  63 | F  M | juxtarenal  infrarenal | -  - | | -  - | -  - | -  - | |  |
| c.5079G>A^n^ | | LB | 1 (1/0) | nd | 1/0 | 64 | F | infrarenal,iliac | 177/188 (1.06) | | 2 | N | 3 | |  |
| ***TGFB2*** | |  | ***2 (2/0)*** |  |  |  |  |  |  | |  |  |  | |  |
| c.272G>A^i^ | | VUS | 1 (1/0) | nd | 1/0 | 58 | M | infrarenal,iliac | 171/175 | | 2 | 3 | 3 | |  |
| c.703G>C | | VUS | 1 (1/0) | nd | 5/2 | 79  66 ^r^  62 ^r^ | M  M  M | infrarenal  infrarenal  infrarenal | 172/184 (1.06)  172/184 (1.06)  - | | 0  0  - | N  3  - | 2,3  1  - | | inguinal hernia |
| ***TGFBR1*** | |  | ***5 (4/1)*** |  |  |  |  |  |  | |  |  |  | |  |
| c.15C>T^i^ | | LB | 1 (1/0) | nd | 3/2 | 44  74 ^r^ | M  M | rupture infrarenal,iliac  infrarenal | 172/181 (1.05)  - | | 0  - | N  - | 3  - | |  |
| c.214A>T | | VUS | 2 (1/1) | nd | 1/1 | 78  69  68 ^r^ | M  M  F | infrarenaal  infrarenal  infrarenal | 178/180  177/185 (1.04)  - | | 0  0  - | N  N  - | 3  3  - | |  |
| c.927G>C | | LB | 1 (1/0) | nd | 1/0 | 66 | M | infrarenal | 176/184 (1.05) | | 9 | 1 | 3 | | inguinal hernia |
| c.1125A>G^m^ | | LB | 1 (1/0) | nd | 4/0 | 75 | F | infrarenal | 174/178 | | 1 | 2 | 3 | |  |
| ***TGFBR2*** | |  | ***3 (2/1)*** |  |  |  |  |  |  | |  |  |  | |  |
| c.1137C>T | | LB | 1 (1/0) | + | 2/1 | 71  72 ^r^ | M  M | infrarenal  ascendens | 178/174  173/168 | | 1  0 | 1,2  N | 1  3 | | inguinal hernia |
| c.1234G>A^l^ | | VUS | 1 (1/0) | + |  | 60  61 | M  M | rupture infrarenal,iliac,popliteal juxtarenal, infrarenal,popliteal | 189/193  183/192 (1.06) | | 1  2 | 1,2,3  1,2 | 2 | | wrist sign |
| **c.1573delA^o^** | | P | 1 (0/1) | de novo | - | 47 | M | infrarenal,iliac,dissection  type B | 189/195 (1.03) | | 7 | N | 3 | | vascular tortuosity  wrist sign |

*Classification of variants: P, pathogenic. LP, likely pathogenic. VUS (variant of unknown clinical significance). LB likely benign.

nd, not determined.

In bold the variants classified as pathogenic or probably pathogenic

** skin signs: scarring, abnormal wide or paper thin

# family: total number of relatives reported to be affected with aortic aneurysm (exludes indexpatient) / the number of relatives confirmed to be affected

^+^ body measurement in cm, ^++^ ratio body length/ arm span < 1.03 (normal) not mentioned

^r^ age relative diagnosed with an aortic aneurysm

^a^ patient with *COL3A1* c.812G>A and *MYLK* c1327C>T

^b^ aortic aneurysm in families of both parents

^c^ mother, father

^d^ patient EFEMP2 c.277G>A and MYLK c.3302A>G variant

^e^ patient with *EFEMP2* c.277G>A, *MYH11* c.5587C>T and *MYLK* c.1327C>T variant

^f^ patient with *EFEMP*2 c.277G>A and *EFEMP2* c.1047C>T variant

^g^ patient with *FBN1* c.59A>G and *MYLK* c.3184G>T variant

^h^ patient *FBN1* c.248-17C>G and *MYH11* c.956A>G variant

^i^ patients with *FBN1* c.1108G>A and *TGFBR1* c.15C>T variant

^j^ patient with *FBN1* c.2260T>C and *TGFB2* c.272G>A variant

^k^ patient *FBN1* c.2895G>A and *MYLK* c.4764G>A variant

^l^ patient with *MYH11* c.4694C>T variant and pathogenic *TGFBR2* c.1234G>A variant

^m^ patient with *MYH11* 5697G>C and *TGFBR1* 1125A>G variant

^n^ patient with *MYLK* c.3583A>G and *MYLK* c.5079G>A variant

^o^ patient with *MYH11* c.5635-7A and the pathogenic *TGFBR2* c.1572delA variant
